# Household Air Pollution and Acute Lower Respiratory Infections in Adults: A Systematic Review

**DOI:** 10.1371/journal.pone.0167656

**Published:** 2016-12-01

**Authors:** Hannah Jary, Hope Simpson, Deborah Havens, Geoffrey Manda, Daniel Pope, Nigel Bruce, Kevin Mortimer

**Affiliations:** 1 Department of Clinical Sciences, Liverpool School of Tropical Medicine, Liverpool, United Kingdom; 2 Malawi-Liverpool-Wellcome Trust Clinical Research Programme, Blantyre, Malawi; 3 College of Medicine, University of Malawi, Blantyre, Malawi; 4 Department of Public Health and Policy, University of Liverpool, Liverpool, United Kingdom; Telethon Institute for Child Health Research, AUSTRALIA

## Abstract

**Introduction:**

Household air pollution from solid fuel burning kills over 4 million people every year including half a million children from acute lower respiratory infections. Although biologically plausible, it is not clear whether household air pollution is also associated with acute lower respiratory infections in adults. We systematically reviewed the literature on household air pollution and acute lower respiratory infection in adults to identify knowledge gaps and research opportunities.

**Methods:**

Ten bibliographic databases were searched to identify studies of household air pollution and adult acute lower respiratory infection. Data were extracted from eligible studies using standardised forms.

**Results:**

From 4617 titles, 513 abstracts and 72 full-text articles were reviewed. Eight studies met the inclusion criteria of which 2 found a significant adjusted increased risk of acute lower respiratory infection, 2 identified a univariate association whilst 4 found no significant association. Study quality was generally limited. Heterogeneity in methods and findings precluded meta-analysis.

**Discussion:**

A systematic review of the literature found limited evidence for an association between household air pollution and risk of acute lower respiratory infection in adults. Additional research, with carefully defined exposure and outcome measures, is required to complete the risk profile caused by household air pollution in adults.

**Registration number:**

CRD42015028042.

## Introduction

Exposure to household air pollution from domestic solid fuel use causes over 4 million deaths per year, and is the most important environmental risk factor for disability adjusted life years worldwide [1]. Household air pollution has been found to increase the risk of childhood acute lower respiratory tract infection (ALRI) by 78% (pooled odds ratio = 1.78 (95% CI 1.45 to 2.18) [2]) and is responsible for half a million deaths in young children every year. Over a third of the world’s population are exposed to high levels of household air pollution, with women and children experiencing the greatest burden [3].

Disease outcomes in adults attributed to household air pollution from solid fuels include chronic lung and cardiovascular disease [1]. Based on the evidence for an association between household air pollution and child ALRI [2], and evidence from ecological and toxicology studies regarding the health effects ambient air pollution in all ages [4], an association between household air pollution and ALRI in adults is biologically plausible but lacks a convincing evidence base. Although the Global Burden of Disease study 2013 incorporated adult ALRI as an outcome for household air pollution exposure, the risk estimate was derived from an integrated exposure-response curve for PM_2.5_ (particulate matter <2.5μm diameter) from ambient and household air pollution, and tobacco smoke which has limitations [1,5]. It is important to determine whether or not there is an increased risk of ALRI associated with household air pollution in adults since the attributable risk is potentially high and the burden greatest in people living in low and middle income countries (LMICs). ALRI is a common cause of hospitalisation among adults in LMICs with high associated morbidity and mortality [6]. Mortality among adults with pneumonia in sub-Saharan Africa is around 10%, with over 50% of deaths seen in people under 35 [7–9].

To ensure that public health interventions are appropriately directed, the true burden of disease needs to be clarified [10, 11]. We systematically reviewed the literature, including studies of any design, to synthesise the evidence base for the relationship between household air pollution exposure from domestic solid fuel use and adult ALRI worldwide. We describe the current literature, identify knowledge gaps and future research opportunities.

## Methods

This systematic review is registered with the Centre for Reviews and Dissemination (Registration number: CRD42015028042). The full protocol is available at http://www.crd.york.ac.uk/prospero.

### Eligibility criteria

Eligibility criteria for studies based on participants, exposures, outcomes and study designs were used to identify studies that provided an effect estimate for household air pollution on adult ALRI in the form of a relative risk, odds ratio (OR) or hazard ratio, or data allowing calculation of an effect estimate. English language papers and non-English language papers (including French and Spanish) where a translation was available were included. Translation was not available for Chinese literature.

### Participants

All adults were eligible, without geographical restriction. Studies exclusively focussed on children (<18 years) were excluded, but to avoid the exclusion of potentially useful data on adults, studies that included low numbers of under 18-year olds within a primarily adult population were included.

### Exposure

Exposure was defined as air pollution from indoor burning of any solid fuels—including wood, charcoal, animal dung, crop residues and coal—for household purposes. We included studies that quantified exposure through direct measurement of specific pollutants, questionnaires regarding exposure history, comparison of groups exposed to types of exposure (e.g. different stove types), or before and after an intervention to reduce exposure. Studies examining outdoor air pollution, occupational exposures, non-fuel combustion sources or non-solid fuels were excluded.

### Outcomes

The primary outcome of interest was ALRI including pneumonia, acute bronchitis or bronchiolitis. However we included studies that defined the outcome as “acute” or specified duration of less than 14 days, even if infection was not confirmed, on the assumption that acute respiratory illnesses in the absence of underlying disease would likely be infectious in origin. Studies reporting illnesses lasting longer than 14 days were excluded, to avoid inclusion of chronic diseases. Studies examining exacerbations of chronic conditions such as asthma or COPD were included if an acute infectious exacerbation was defined. Studies that did not distinguish between upper respiratory tract infections and ALRI were excluded.

### Study designs

Individually- and cluster-randomised control trials; controlled before-and-after trials; cohort studies; case-control studies and cross-sectional surveys were included.

### Search strategy

The databases listed in Box 1 were searched on 17/12/2015 using the search terms and Boolean phrases listed in Box 2. An example search strategy is shown in Box 3. Searches were limited to papers published from 01/01/1960 onwards, and to human studies. Identified papers were imported into EndNote X7.

**Box 1. Databases searched**

- MEDLINE (OVID)
- Scopus (including EMBASE)
- Web of Science
- CINAHL (Ovid)
- Global Health (Ovid)
- Cochrane Central Register of Controlled Trials (CENTRAL)
- Database of Abstracts of Reviews of Effects (DARE)
- Latin American and Caribbean literature (LILACS)
- SciELO
- African Index Medicus

**Box 2. Search terms and Boolean phrases used**

*Outcomes* (**OR**): “respiratory infection*”, “respiratory tract infection*”, pneumonia, “respiratory illness*”

**AND**

*Exposures/Interventions* (**OR**): “household air”, “indoor air”, biomass,*smoke, fuel*, *stove*.

**Box 3. Example search strategy for Medline (OVID)**

1. “respiratory infection*”.mp,tw.
2. “respiratory tract infection*”.mp,tw.
3. pneumonia.tw,mp.
4. “respiratory illness”.tw,mp.
5. 1 or 2 or 3 or 4
6. “household air”.tw,mp.
7. “indoor air”.tw,mp.
8. biomass.tw,mp.
9. smoke,tw,mp.
10. stove.tw,mp.
11. fuel.tw,mp.
12. 6 or 7 or 8 or 9 or 10 or 11
13. 5 and 12

Following removal of duplicates, titles of identified papers were reviewed for eligibility by two authors. The first 20% were all reviewed in duplicate by two independent authors to check for agreement. The remaining 80% were reviewed by one author with 10% of these being cross-checked by a second author to ensure ongoing agreement.

Abstracts of the selected titles and then full text of the selected articles were reviewed for eligibility, according to the selection criteria. Abstracts and full text articles were all reviewed in duplicate by two independent authors—HJ reviewed all abstracts and full text articles and the second review was performed by either HS, DH or GM. Where discordant decisions were not resolved by discussion, the decision was made by a third author. If the full text article was not available in English, translation was sought to determine eligibility.

Alternative sources were also searched for eligible studies: www.who.int/trialsearch, www.clinicaltrials.gov and EAGLE (European Association for Grey Literature Exploitation, www.opengrey.eu). Reference lists of all selected papers were reviewed for any potentially eligible titles. For studies where relevant details were not available, authors were invited to supply information if their contact details were available.

### Data extraction

Data were extracted from the selected papers using a previously piloted data extraction form, from which a summary table and narrative synthesis were produced. Where effect estimates were not provided, these were calculated from the published data.

### Quality assessment

Studies that quantified exposure by direct household or personal measurement of specific pollutants were considered to provide a higher quality of exposure assessment than those that relied on self-reported exposure. Studies using prospective assessment of ALRI by a physician or trained health care worker using predefined criteria or diagnostic investigations were considered to be of higher quality than those reliant of self-reported episodes of ALRI.

### Risk of bias assessment

Risk of bias was assessed using the Liverpool Quality Assessment Tool, which reviewed the study methodology in four domains—subject selection, exposure assessment, outcome assessment and adjustment for confounders—and assigned quality ratings (low, moderate or high risk of bias) [12]. We adopted this instead of the Cochrane Collaboration's tool for assessing risk of bias due to the variety of study designs identified. Publication bias was not assessed due to the small number of papers identified.

### Data analysis

Methodological heterogeneity in exposure and outcome assessment precluded a meta-analysis, but a narrative summary of the selected papers and a summary of findings table were produced.

## Results

**Fig 1:**
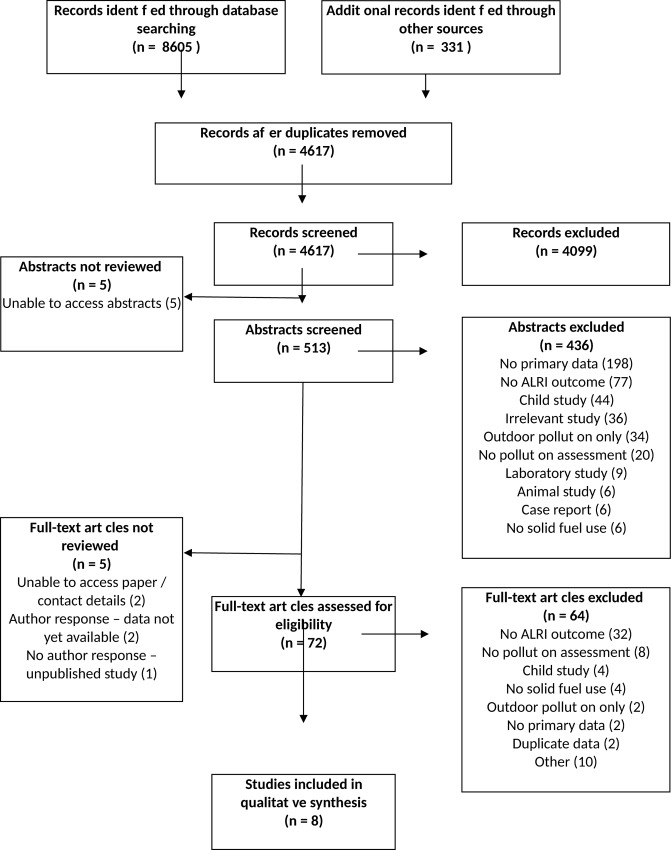
PRISMA Flow Chart A flow chart depicting the inclusion and exclusion of identified studies

Database searches identified 8605 records and 331 records were identified from the alternative sources set out in the methods. After removal of duplicates 4617 records remained. The number of titles, abstracts and papers reviewed was 4617, 513 and 72 respectively, as summarised in the PRISMA flowchart (Fig 1), with the reasons for exclusion at the abstract and full text stages (see S1 File for PRISMA Checklist). Agreement between authors was 95–96% for the first 20% of titles reviewed, and 95–98% for the 10% overlap of remaining titles. It was not possible to locate 5 selected abstracts [13–17] and 2 selected full text papers—both published in Chinese [18, 19]—for review. Two authors were contacted for further details about their recent or ongoing studies identified at www.clinicaltrials.gov. One study is a prospective cohort study of febrile adults in Tanzania for which recruitment has been completed but the data are not yet available (NCT01947075). The second study is a randomized trial of smoke reduction interventions in Native American populations, but we were unable to obtain further details (NCT02240069). In addition, we are conducting a case-control study of the effects of air pollution exposure and chronic respiratory disease on pneumonia risk in Malawian adults [20].

Ten papers met the criteria for inclusion; data from 2 of these were duplicated in other selected papers [21, 22], and therefore not extracted. The remaining 8 papers included 4 cross-sectional [23–26], 2 cohort [27, 28] and 2 case control studies [29, 30]. The articles covered study locations from 8 countries across 4 continents. The largest study was a retrospective cohort study from China, which reported on the causes of death in 42,422 farmers [28]. The main findings of all included studies are summarised in Table 1, which demonstrates the heterogeneity of study participants, exposure definitions and outcome definitions.

**Table 1:** Summary of findings table for four studies investigating the effects of household air pollution on adult acute lower respiratory tract infections.

Study	Sample selection	Sample size	Subject description	Exposure	Outcome	Adjustment for confounders	Effect size
**COHORT STUDIES**							
Ezzati, 2001 [27]. Kenya, rural. Conducted 1996–1999.	55 randomly chosen households. Response rates of household members not stated.	229 individuals.	47.6% 5–14 years. 52.4% 15–49 years. Age groups treated as one category, but ALRI more common in the latter. 55% female.	Continuous measurement of PM_10_ in households for 14–15 hours a day for 200 days.	ALRI (including bronchitis, pneumonia and broncho-pneumonia diagnosed by a nurse who visited all households every 1–2 weeks for 2 years, and examined anybody reported to have respiratory symptoms.	Adjusted for age, sex, smoking, village, and household occupancy. No adjustment for socioeconomic status, but homogenous group of participants.	Adjusted logistic regression, OR (95% CI, p value): Reference category: <200μg/m^3^ PM10^**•**^. 200–500 μg/m^3**†**^: 1.65 (0.5–5.45, 0.41). 500–1000 μg/m^3**†**^**:** 1.87 (0.6–5.71, 0.27). 1000–2000μg/m^3**†**^**:** 2.74 {0.93–8.12, 0.07). 2000–4000μg/m^3**†**^**:** 3.28 (1.09–9.85, 0.03). 4000–7000 μg/m^3**†**^: 3.21 (1.01–10.24, 0.05). >7000 μg/m^3**†**^: 7.10 (2.26–22.32, 0.001).
Shen, 2009 [28]. China, rural. Conducted 1992–1996.	Identified from local administrative records.	44,850 individuals identified. 42,422 followed up to endpoint.	All famers in 4 communes born between 1917 and 1951 and living in Xuanwei on 1/1/1976. 49% female.	Principal use of smoky or smokeless coal, and presence of chimney, assessed by standardised questionnaire.	Death from pneumonia obtained from public records of death certificates, which were completed by physicians.	Adjusted for annual coal use, stove improvement, smoking, years of cooking, education, house size and occupancy, coal mining, COPD and time spent indoors.	Cox proportional hazards model, HR (95%CI, p value). Stove improvement^**†**^ vs no stove improvement^**•**^. Men: 0.49 (0.31–0.78, 0.002). Women: 0.53 (0.32–0.88, 0.014). Smokeless coal^**†**^ vs smoky coal^**•**^. Men: 1.52 (0.98–2.36, 0.060). Women: 1.44 (0.90–2.31, 0.129).
**CASE CONTROL STUDIES**							
Loeb, 2009 [29]. Canada, urban. Conducted 2002–2005.	Cases were patients who presented to emergency departments. Not stated if all consecutive patients recruited. Controls recruited contemporaneously using random-digit dialing. Response rates not reported.	717 cases and 867 controls.	Cases: pneumonia patients, 40% female, age >65 (mean 79). Controls: unmatched healthy community controls, 68% female, age >65 (mean 74). The same geographical restrictions were used for both groups.	Recall of fireplace use in previous 12 months, assessed by structured questionnaire. No quantification of exposure.	Pneumonia, based on appropriate clinical criteria plus radiographic findings, all performed by physicians.	Backward-stepwise logistic regression performed adjusting for multiple variables but fireplace use not included in the final model so only unadjusted results reported.	Unadjusted logistic regression, OR (95%CI, p value): Fireplace use^**†**^ vs. no fireplace use^**•**^. 0.69 (0.54–0.87, 0.002).
Figueroa, 2012 [30]. Mexico, urban. Conducted 2000–2007.	Retrospective review of hospital records and social work department records. Implied that all consecutive records included but not implicitly state.	948 cases and 1305 controls.	Cases: bacterial pneumonia, 42% female, age >18 (mean 55). Controls: otolaryngeal patients, 38% female, age >18 (mean 32).	Past or current exposure to wood smoke in the home, based on retrospective review of secondary source documents. No quantification of exposure.	Bacterial pneumonia, based on retrospective review of hospital records by specialists using a standardised format and clinical definition of pneumonia.	Adjusted for age, gender, occupational exposures, Type 2 Diabetes and household ventilation. No adjustment for socioeconomic status or outdoor exposures.	Logistic regression, OR (95% CI, p value): (unadjusted results calculated from raw data). Current wood smoke exposure^**†**^ vs No current wood smoke^**•**^. Unadjusted: 2.62 (1.78–3.86, <0.0001). Past wood smoke exposure^**†**^ vs No past wood smoke^**•**^. Unadjusted: 2.14 (1.89–2.55, <0.0001). Adjusted: 1.1 (0.9–1.4, 0.5).
**CROSS SECTIONAL STUDIES**							
Shrestha, 2005 [23]. Nepal, rural (81%) & urban (19%). Conducted 2003–2004.	Households randomly selected. Household member response rates not stated.	98 households.168 respondents.	94% female, mean age 36 years (S.D. 16.7), minimum age not stated.	Use of “unprocessed fuel” (solid bio-fuels) vs “processed fuels” (gas / kerosene), assessed by questionnaire.	ALRI, based on physician examination and review of symptoms but no definition stated. Not stated whether retrospective diagnoses included.	Adjusted for smoking* and age**	Unadjusted OR (95% CI): Unprocessed fuel^**†**^ vs processed fuel use^**•**^. 2.69 (0.76–9.52). Adjusted OR (95% CI): Unprocessed fuel^**†**^ vs processed fuel use^**•**^. *2.77 (0.77–10.00). **2.63 (0.74–9.31).
Kilabuko, (2007) [24]. Tanzania, rural. Conducted 2004.	Random sampling of household. No refusal rates for household members stated.	100 households.390 participants.	No demographics of participants stated, but includes age 5 and over. “Chief cooks” were mainly wives of household heads, so assumed predominantly adult but not necessarily.	“Chief cooks” compared to other household members. No explanation of how chief cooks were chosen or defined. Likely significant overlaps between 2 groups. No quantification of exposure.	ARI, defined as household member reported cough with rapid breathing, assessed by questionnaire. No recall period defined.	No adjustment for confounders.	Unadjusted OR (95% CI, p value) calculated from raw data. Chief cook^**†**^ vs other household members^**•**^. 3.76 (2.19–6.48, <0.0001).
Stanković 2011 [25]. Serbia, urban. Conducted 2008.	Individuals recruited from a health centre when attending for health checks. No description of how the sample was selected. Response rates not reported.	1082 participants.	All female, age 20–40.	Self-reported ‘use of biomass fuels’, assessed by questionnaire. No quantification of exposure.	Self-reported “doctor diagnosed pneumonia’ or ‘doctor diagnosed bronchitis’ in their life time, assessed by questionnaire.	Adjusted for age, education, family history of respiratory illness and outdoor air pollution. Not explicit whether adjusted for environmental tobacco smoke, home dampness or pets. Not adjusted for occupational exposures, comorbidities or socioeconomic factors.	Adjusted logistic regression, OR (95% CI). Biomass use^**†**^ vs no biomass use^**•**^. Bronchitis: 0.91 (0.71–1.15). Pneumonia: 0.99 (0.80–1.22).
Taylor, 2012 [26]. Sierra Leone, rural and peri-urban. Conducted 2011.	Participants randomly selected from all eligible individuals in the study area in 16 community strata, using stratified sampling. Response rates not stated.	520 participants.	All female, age 15–45.	Kitchen location, type of fuel normally used and number of hours spent in the kitchen, assessed by questionnaire.	ARI, defined as self-reported cough followed by rapid breathing, assessed by questionnaire with a 2-week recall period.	Adjusted for age, marital status, kitchen type, smoking, housing type and number of rooms. Not adjusted for other pollution exposures comorbidities or socio-economic factors.	Logistic regression, OR (95% CI, p value): Wood^**†**^ vs charcoal^**•**^. Adjusted: 1.14 (0.71–1.82, 0.580). Kitchen inside the main house^**†**^ vs separate kitchen^**•**^. Adjusted: 0.68 (0.38–1.24, 0.210). Effect of spending 4–6 hours^**†**^ in kitchen or >7 hours^**†**^ vs <3 hours^**•**^. Unadjusted: 1.31 (0.88–1.94, 0.179) and 2.40 (0.86–6.72, 0.094), respectively.

PM_10_ = Particulate Matter < 10μm diameter; ALRI = Acute Lower Respiratory Infection; ARI = Acute Respiratory Infection; COPD = Chronic Obstructive Pulmonary Disease; OR = Odds Ratio; HR = Hazard Ratio; CI = Confidence Interval

The exposure and comparator used in each analysis are notated by ^†^ and ^•^ respectively

Most studies were based in rural settings and recruited participants from communities. There were 3 exceptions, which were hospital or health-centre based studies, conducted in urban areas of Canada, Mexico and Serbia [25, 29, 30].

Most studies predominantly or exclusively recruited females (accounting for 48–100% of participants within studies). In the study from Tanzania, the cooks were mainly female but the gender and age of the unexposed group were not reported [24].

Except for the study from Canada, which restricted recruitment to individuals over 65 [29], and the study from China [28], in which deaths occurred between 25 and 80 years of age, all studies recruited younger adults. Five studies restricted participants to adults over 18, whilst the 3 remaining studies—from Sierra Leone, Tanzania and Kenya—included younger participants but were conducted in predominantly adult populations.

One study evaluated coal use (Shen *et al*) and the 7 others evaluated biomass fuel use, predominantly wood. Four studies measured levels of household pollutants directly, but only 1 presented an estimate for the effect of pollutants on ALRI risk. In Kenya, Ezzati *et al*. measured particulate matter <10μm diameter (PM_10_) for 14–15 hours per day for 200 days in 55 households, and demonstrated a dose-response relationship between exposure to PM_10_ and ALRI risk [27]. The authors report that this finding was supported by analysis with a continuous-exposure variable and inverse-quadratic relation, although have not published these results. All studies monitoring air pollution levels measured PM_10_ rather than PM_2.5_. This may misrepresent the true harmful exposure, as smaller PM_2.5_ particles are inhaled deeper in to the lungs than PM_10_ and are therefore thought to be more pathogenic [31].

The 7 studies that did not provide an effect estimate for measured pollutants all used questionnaire data to classify exposure to household air pollution, based on a variety of self-reported measures including fuel or stove type, quantification of fuel use, ventilation, cooking frequency and location.

Two studies defined the outcome using prospective health care worker diagnosis of ALRI; in Canada, hospitalised cases had symptoms or signs, and radiological changes consistent with pneumonia [29]; in Kenya, the outcome was defined as nurse-diagnosed ALRI following review and physical examination at 1–2 weekly visits over a 2 year period [32]. A third study, from Nepal, included a physician’s review to diagnose ALRI, but no further definition was provided and it is unclear whether retrospective diagnoses were included [23]. The study from China used retrospective review of death certificate records, which had been completed by physicians on the basis of clinical and radiological investigations [32]. Similarly, the study in Mexico used a retrospective review of hospital records by specialists to identify cases [30]. The remaining studies used self-reported symptoms or past diagnoses, with varying recall periods, to define the outcome [24–26].

Six of the 8 studies made some adjustment for potential confounders in their analysis of the effect of household air pollution on ALRI [23, 25, 26, 28, 30, 32], but several studies failed to make adjustments for important potential confounders, such as economic status and smoking.

**Fig 2:**
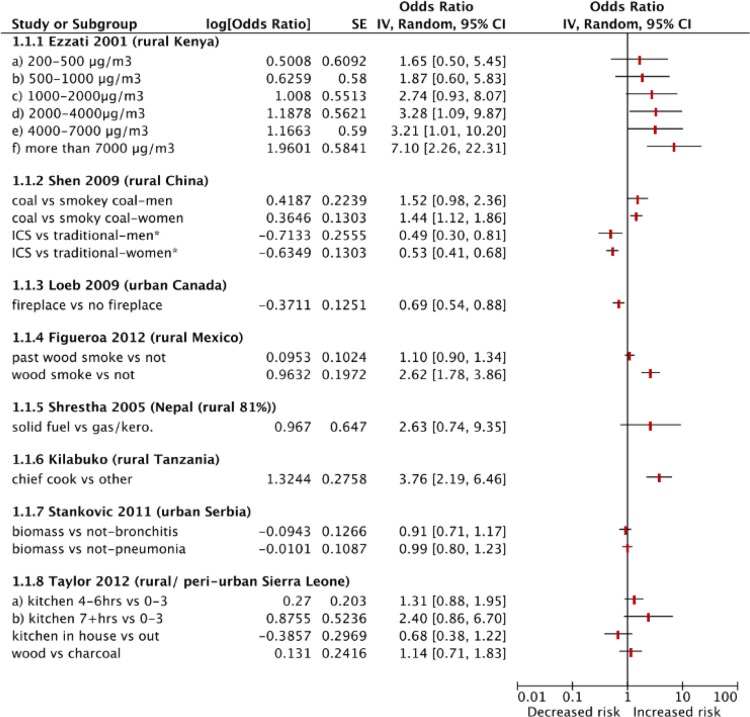
Forest Plot A forest plot showing the Odds Ratio (and lower and upper 95% Confidence Intervals) for different exposures and outcome in the included studies. Adjusted results are shown where available. Key: ICS: improved cookstove; kero: kerosene; *indicates that the study is assessing for the protective effect of an intervention.

The effect estimates quantified by the studies are shown in Table 1 and in a Forest Plot (Fig 2). There were inconsistent findings across the studies. Whilst the 2 cohort studies have marked differences in study design, they demonstrated statistically significant harmful effects of household air pollution exposure on ALRI after adjustment for confounders. A dose-dependent relationship between PM_10_ and ALRI was detected in Kenya, (OR = 7.10, (95% CI 2.26 to 22.32) for exposure to >7000μg/m^3^ PM_10_ (highest exposure category) vs. 200–500 μg/m^3^ PM_10_) [32], and a reduction in risk of pneumonia deaths was observed in participants using an improved coal stove instead of a traditional coal stove (OR = 0.49 (95% CI 0.31 to 0.80) and 0.53 (95% CI 0.32 to 0.88)) in Chinese men and women respectively [28]. The studies from Mexico and Tanzania both reported harmful effects of household air pollution exposure in univariate analysis [24, 30], but these were no longer significant after adjustment for confounders in the Mexican study. The studies from Nepal, Serbia and Sierra Leone did not identify a significant association between household air pollution and risk of ALRI [23, 25, 26]. The study from Canada found that fireplace use was associated with a reduction in the risk of pneumonia, although this was unadjusted [29].

All of the case-control and cross-sectional studies were found to be at high risk of bias in at least 2 out of the 4 domains assessed, leading to an overall assessment of “moderate/high” or “high” risk of bias. Exposure assessments were weak. The cohort studies were of a generally higher standard, although their risk of bias was moderate in at least 2 out of 4 domains.

## Discussion

This paper synthesises the current evidence base for the relationship between household air pollution and adult ALRI, identifying 8 eligible studies that have quantified this relationship. Despite a paucity of available studies, the available data provides some evidence for an increased risk of adult ALRI from exposure to household air pollution. The review has highlighted disparities between the findings of the studies, although these are unsurprising given the methodological heterogeneity seen. Methodological limitations were also noted, including poor exposure and outcome classifications, and potential biases including selection and recall biases. Although suggestive of an association, the current evidence is not sufficient to make a direct assessment of the potential impact of household air pollution on adult ALRI.

Methodological differences in exposure classification are a likely source of variation between study findings in this review. Indirect classification of exposure to household air pollution was common, and may have resulted in exposure misclassification. Accurate measurement of exposure to pollutants such as PM_2.5_ is challenging in the field. Monitoring is becoming more practical, with a variety of devices now available, which will benefit future population studies of health impacts such as ALRI. Exposure, however, needs to be measured at repeated intervals to accurately classify levels for diseases with a long latency period from defined exposures and this remains a challenge.

With regards to major sources of bias and error, the quality of studies identified was varied. Two studies used retrospective records of patients, and none of the prospective studies reported response or refusal rates of participants, so may have been subject to selection bias. Several studies used some form of random sampling but the cross-sectional study from Serbia only recruited women who attended a health centre so the findings are not generalisable to the wider population. The case-control study from Mexico used patients with otolaryngeal disease as controls, but as household air pollution can also be a risk factor for this outcome [3], this may have biased the findings towards the null. Only 1 study directly measured exposure to household pollutants [32]. All other studies relied on questionnaire responses regarding exposures, which are prone to recall bias, with varying recall periods and often poor definitions of exposure. Similarly, several studies relied on potentially biased and inaccurate recall of previous illness; outcome misclassification may have diluted the true effect. Three studies used prospective assessment of patients by a health-care worker for the outcome classification, although the quality of definitions used varied. Two studies made no adjustment for confounders, and other studies failed to adjust for some important confounders such as smoking, and so their findings may be misleading. Four studies were cross-sectional and are therefore unable to establish temporality between exposure and outcome.

Although the literature was previously reviewed as part of the Comparative Risk Assessment conducted for the GBD 2010 [33], this review provides an update, adding 5 studies to the evaluation. This review also provides a narrative summary of the current evidence which was not previously available, and a critical appraisal of methodological limitations. The review included a comprehensive search of published bibliographic databases—including databases from Africa and Latin America—and available grey literature, including contact with known researchers in the field undertaking relevant research. A limitation of this review is that we were unable to include literature published in Chinese. Due to the paucity of evidence, publication bias was not assessed.

Diseases associated with household air pollution disproportionately affect those living in poverty, who rely on solid fuels to meet their energy needs [34]. To consolidate GBD estimates—which currently use modelled data from other sources of air pollution—we set out to quantify the association between household air pollution and adult ALRI. Whilst it is likely that the burden of disease from household air pollution in adults includes ALRI, the published literature does not provide definitive evidence of this. Further research, using robust direct exposure measurement and accurate classification of outcome, is required to improve the evidence base. Exposure to household air pollution is preventable but resources are limited in low-income populations with competing health priorities: with high quality evidence of the true scale of the problem and cost-effectiveness of interventions, resources to reduce the global burden of disease can be effectively allocated.

## Supporting Information

S1 File PRISMA Checklist. PRISMA Checklist.
(DOC)
